# Adipose tissue hyperplasia with enhanced adipocyte-derived stem cell activity in *Tc1*(*C8orf4*)-deleted mice

**DOI:** 10.1038/srep35884

**Published:** 2016-10-24

**Authors:** Hayoung Jang, Minsung Kim, Soyoung Lee, Jungtae Kim, Dong-Cheol Woo, Kyung Won Kim, Kyuyoung Song, Inchul Lee

**Affiliations:** 1 Asan Institute for Life Sciences, Asan Medical Center, University of Ulsan College of Medicine, Seoul, Korea; 2 Department of Biochemistry and Molecular Biology, Asan Medical Center, University of Ulsan College of Medicine, Seoul, Korea; 3 Department of Pathology, Asan Medical Center, University of Ulsan College of Medicine, Seoul, Korea

## Abstract

Adipose tissue hyperplasia with increased number of adipocytes is implicated in a protective rather than deleterious effect on obesity-associated metabolic disorder. It is poorly understood how the adipose tissue cellularity is regulated. *Tc1* is a gene of vertebrates that regulates diverse downstream genes. Young *Tc1*-deleted mice fed on standard chow diet show expanded adipose tissue with smaller adipocytes in size compared to wild type controls, representing adipose tissue hyperplasia. *Tc1*^−/−^ mice show enhanced glucose tolerance and reduced serum lipids. Adipocyte-derived stem cells (ADSCs) from *Tc1*^−/−^ mice show enhanced proliferative and adipogenic capacity compared to wild type controls, suggesting that the adipose hyperplasia is regulated at the stem cell level. PPARγ and CEBPα are up-regulated robustly in *Tc1*^−/−^ ADSCs upon induction for adipogenesis. Wisp2 and Dlk1, inhibitors of adipogenesis, are down-regulated in *Tc1*^−/−^ ADSCs compared to controls. *Tc1*-transfected NIH3T3 cells show higher β-catenin reporter signals than vector transfected controls, suggesting a role of canonical Wnt signaling in the Tc1-dependent adipose regulation. Our data support that Tc1 is a novel regulator for adipose stem cells. Adipose tissue hyperplasia may be implicated in the metabolic regulation of *Tc1*^−/−^ mice.

Adipose tissue plays a critical role in the systemic and metabolic homeostasis^1^,^2^. It is developed from precursor cells known as adipocyte-derived stem (stromal) cells (ADSCs) or adipose tissue-derived mesenchymal stem cells (AT-MSC) under tight regulation^3^. PPARγ, CEBPs, and Wnt signaling have been implicated in the adipogenic regulation^4^,^5^,^6^.

Obesity is increasing in an epidemic manner in most countries, and associated metabolic disorders pose serious public health problems. Obesity has largely been attributed to food overconsumption and decreased physical activity resulting in the expansion of white adipose tissue (WAT). WAT expansion occurs by two distinct mechanisms: hypertrophy (increase in adipocyte size) and hyperplasia (increase in adipocyte number). It has been suggested that obesity-associated deleterious complications may depend on the mode WAT expansion. Limited expandability of hypertrophic obesity has been associated with dyslipidemia^7^, insulin resistance^8^, and type 2 diabetes (T2DM)^9^,^10^,^11^,^12^. On the other hand, it was suggested that subcutaneous hyperplastic obesity might have a protective role against such metabolic disturbances^13^,^14^. Transplantation of subcutaneous adipose tissue in lipodystrophic mice cures hepatic steatosis and insulin resistance^15^. Pharmacological induction of adipose tissue proliferation by thiazolidinediones is followed by restoration of insulin sensitivity in humans^16^,^17^. Genetic predisposition may play a role for individual patterns of obesity. However, the cellular and molecular mechanisms regulating adipocyte size, number, and metabolic regulation *in vivo* remain largely unknown.

*TC1*(*C8orf4*) is a well conserved gene of vertebrates. It has been implicated in various carcinomas and hematological malignancies^18^,^19^,^20^,^21^,^22^,^23^,^24^. It is associated with poor prognosis of cancer patients^21^. TC1 appears to have diverse cellular and systemic regulatory roles. The expression is enhanced by pro-inflammatory cytokines, heat shock, and various cellular stresses^25^,^26^. It enhances Wnt/β-catenin signaling in cancer cells by relieving antagonistic activity of Cby^27^. It also activates endothelial cells enhancing classical NF-κB signaling^28^. In zebrafish embryo, which has 2 homologues, they are expressed in association with blood vessels or hematopoietic cells^28^.

We have reported previously that *Tc1*-deleted mice show increased white cells in peripheral blood and enhanced hematopoietic activity^29^. Here, we report a novel biological role of Tc1 in adipose tissue, stem cell, and metabolic regulation.

## Results

### Young *Tc1* ^−/−^ mice are heavier than wild type

**Figure 1:**
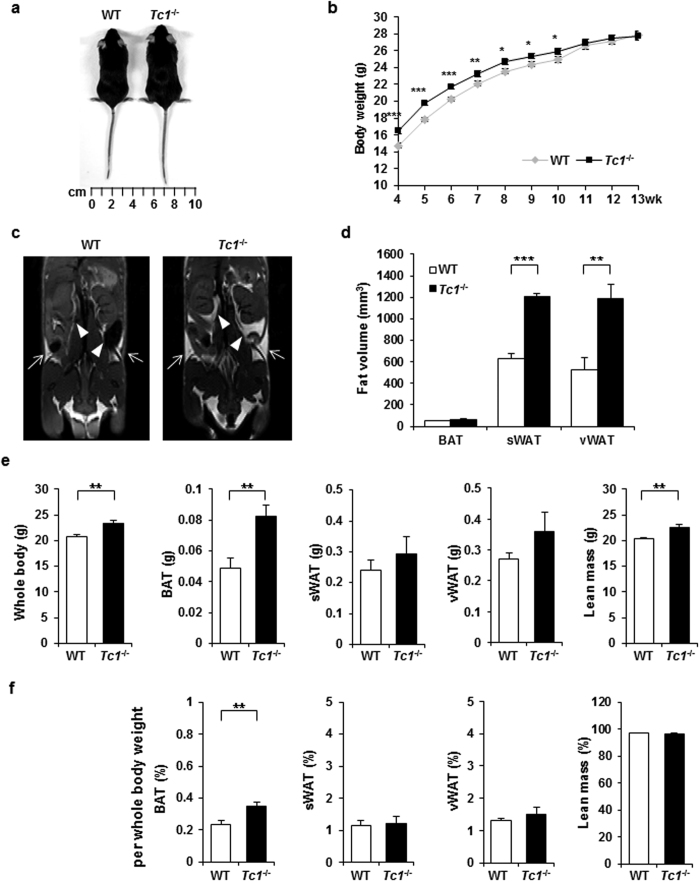
Adipose hyperplasia in Tc1^−/−^ mice. (**a**) Representative photograph of 9 week old wild type (WT) and Tc1^−/−^ mice. (**b**) Body weight of Tc1^−/−^ and WT male mice between 4 and 13 weeks of age. WT, *n* = 44~65 mice per each group; Tc1^−/−^, *n* = 33~113 mice per group. (**c**) Representative magnetic resonance imaging (MRI) sections showing body fat distribution of 10 week old male WT and Tc1^−/−^ mice. Subcutaneous white adipose tissue (sWAT) shown by arrows, and visceral white adipose tissue (vWAT) by arrowheads. (**d**) Calculated body fat volume by MRI analysis. Data represent mean ± s.d. from 4 male, 10 week-old *Tc1*^−/−^ mice, and 4 wild type control mice. (**e**) Weights of whole body, BAT, sWAT, vWAT, and lean body mass of male *Tc1*^−/−^ mice and age- and sex-matched wild type (10 week old, n = 8 each group). (**f**) Percentage per whole body weight calculated from data above (**e**). *p < 0.05, **p < 0.01, ***p < 0.001.

The animals were fed standard chow diet *ad libitum*. *Tc1*^−/−^ mice were significantly heavier than wild type controls until 10 weeks (Fig. 1a,b). Four week old *Tc1*^−/−^ mice were heavier by 11.88% in average, but the gap decreased gradually until 13 weeks when they were similar in weight with wild types. Young *Tc1*^−/−^ mice did not eat more than wild types did (Supplemental Fig. S1), suggesting that the disparity of weight in youth was not dependent on eating pattern. From 13 weeks on, they tended to eat slightly more than wild types.

To investigate the adipose tissue volume *in vivo*, whole body MRI of 10 week old *Tc1*^−/−^ and wild type control mice was analyzed. Both subcutaneous white adipose tissue (sWAT) and visceral WAT (vWAT) were evidently expanded in *Tc1*^−/−^ mice compared to wild type controls (Fig. 1c). The calculated volumes of sWAT and vWAT were increased significantly in *Tc1*^−/−^ compared to control mice (Fig. 1d).

### Adipose tissue hyperplasia in *Tc1* ^−/−^ mice

sWAT, vWAT, and brown adipose tissue (BAT) were dissected separately from 10 week old *Tc1*^−/−^ mice and control mice. The whole body weight and BAT of *Tc1*^−/−^ mice were heavier than control mice (Fig. 1e). sWAT and vWAT were also heavier, but the differences were marginal. The lean body mass also increased in *Tc1*^−/−^ mice proportional to the total body weight (Fig. 1e,f). As the percentages per whole body weight were compared, the percentage of BAT was significantly higher in *Tc1*^−/−^ mice than in wild type (Fig. 1f). No significant difference of body weight, adipose tissue, and lean body mass were shown in 23 week old *Tc1*^−/−^ mice compared to controls (Supplemental Fig. S2).

**Figure 2:**
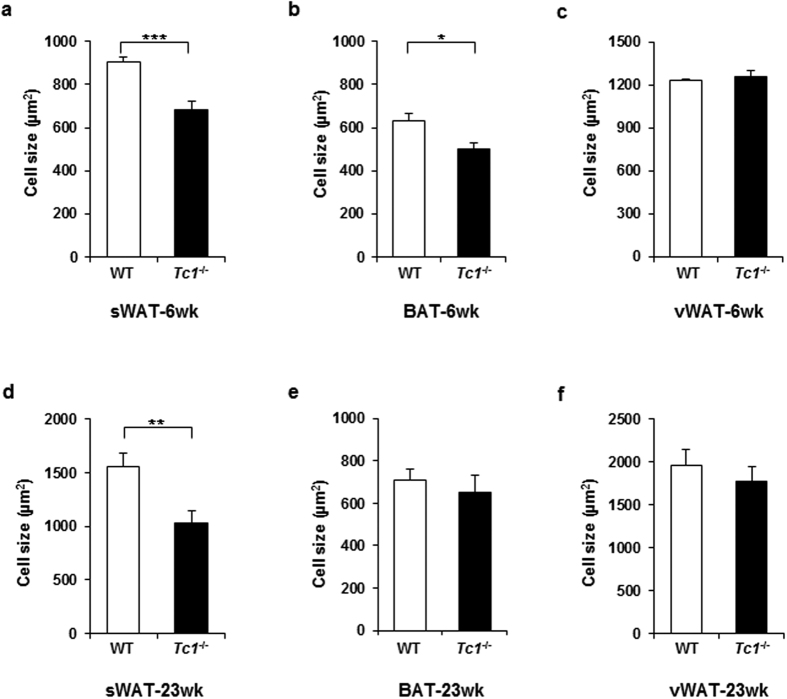
Smaller adipocyte size in Tc1^−/−^ mice. (**a–c**) Adipocyte size from sWAT, BAT, and vWAT of 6 week old, male *Tc1*^−/−^ mice and age- and sex-matched wild type mice (n = 6 each group). Adipocytes were analyzed using Image J software (NIH, MD, USA) as described in Methods. (**d–f**) Adipocyte size of sWAT, BAT, and vWAT from 23 week old, male *Tc1*^−/−^ mice and wild type mice (n = 5 each group). *p < 0.05, **p < 0.01, ***p < 0.001.

To investigate the nature of expansion, adipose tissues were analyzed histologically. No evident difference was noted in the vascular development or mononuclear infiltration between *Tc1*^−/−^ and wild type mice. Interestingly, sWAT and BAT adipocytes of 6 week old *Tc1*^−/−^ mice were significantly smaller in size compared to wild type, representing adipose tissue hyperplasia with increased adipocyte numbers per volume (Fig. 2a,b). vWAT adipocytes were not different in size (Fig. 2c). Adipocytes of aged mice were larger than those of young animals (Fig. 2d–f). sWAT adipocytes of 23 week old *Tc1*^−/−^ mice were also significantly smaller in size compared to wild type controls (Fig. 2d), suggesting that the hyperplasia was maintained in aged animals. BAT and vWAT adipocytes were not significantly different in size in aged mice (Fig. 2e,f).

### Reduced serum lipids and enhanced glucose tolerance in *Tc1* ^−/−^ mice

**Figure 3:**
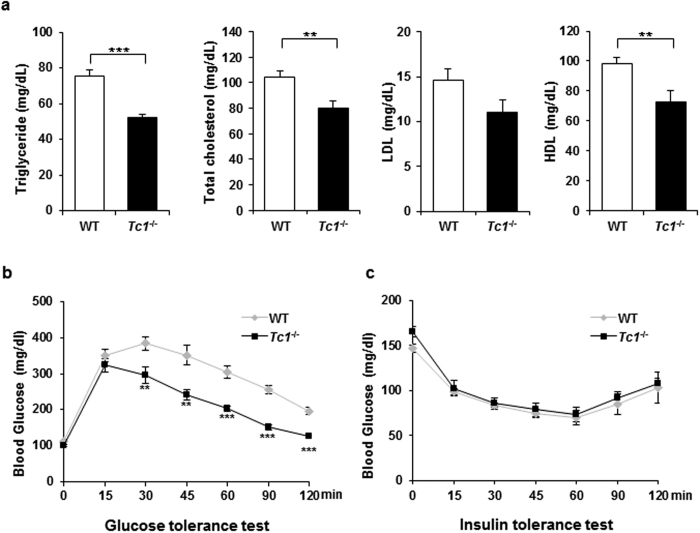
Serum lipids and glucose tolerance test. (**a**) Fasting serum levels of triglyceride, total cholesterol, low-density lipoprotein (LDL), and high-density lipoprotein (HDL) from 10 week old mice. Data represent mean ± s.d. of 6 wild type and *Tc1*^−/−^ mice, respectively. (**b**) Glucose tolerance test. Mice are fasted overnight, and injected with 2% glucose solution intraperitoneally, 2 g/kg body weight per animal. Blood glucose measured as indicated after the injection. Data represent mean ± s.d. of 10 week old wild type and *Tc1*^−/−^ mice (n = 6), respectively. (**c**) Insulin tolerance test. Animals are fasted for 5 hours (n = 4 each), and injected with recombinant insulin (Humulin, Eli Lily), 1unit/kg, intraperitoneally. Blood glucose is measured similarly. Data represent mean ± s.d. of 10 week old wild type and *Tc1*^−/−^ mice (n = 4), respectively. **p < 0.01, ***p < 0.001.

**Figure 4:**
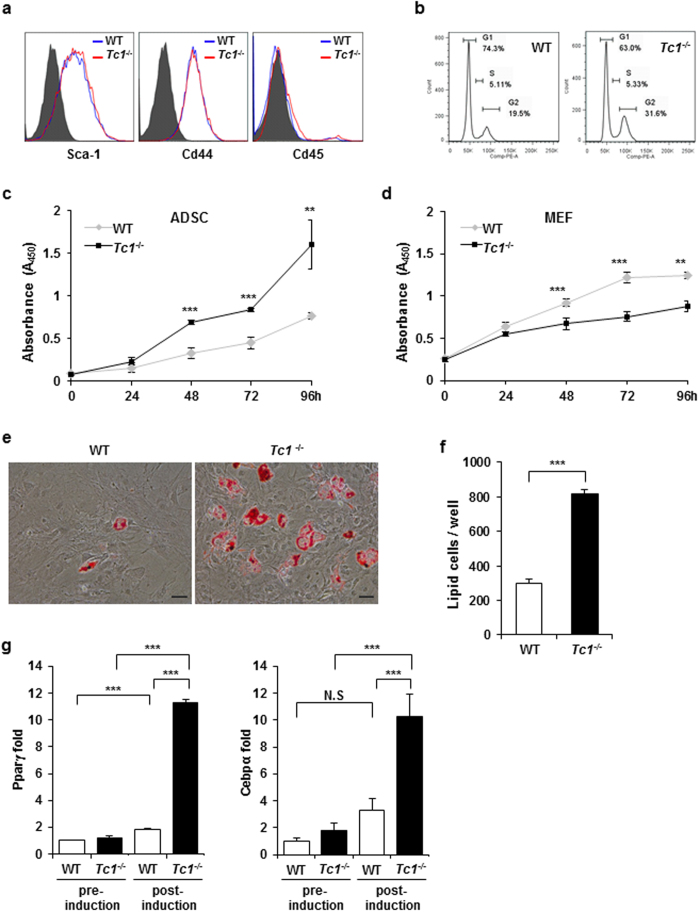
Adipocyte-derived stem cell (ADSCs) proliferation and *in vitro* adipogenesis. (**a**) Representative flow cytometric analysis of ADSCs isolated from inguinal sWAT of wild type and *Tc1*^−/−^ mice, 8 week old. Expressions of Sca-1, CD44, and CD45 are shown with unstained negatives. (**b**) Cell cycle analysis of wild type and *Tc1*^−/−^ ADSCs using propidium iodide DNA staining. Cell populations in G1, S, and G2 phases are shown. (**c**) Cell proliferation of wild type and *Tc1*^−/−^ ADSCs analyzed using WST-1 proliferation assay kit (Roche). Data represent mean ± s.d. of 4 independent experiments. Statistically different data are indicated. (**d**) Cell proliferation of wild type and *Tc1*^−/−^ mouse embryonal fibroblasts (MEFs). Data represent mean ± s.d. of 4 independent experiments. (**e**) Representative photographs of Oil Red O-stained wild type and *Tc1*^−/−^ ADSCs induced for adipogenesis. (Oil Red O staining, bars 100 μm). (**f**) Oil Red O-positive cells/well in 6 well plates. Data represent mean ± s.d. of 4 independent experiments. (**g**) Real-time PCR for Pparγ and Cebpα in wild type and *Tc1*^−/−^ ADSCs before and 24 h after adipogenic induction. Data represent mean ± s.d. from 3 independent experiments. *p < 0.05, **p < 0.01, ***p < 0.001.

Adipose tissue hyperplasia has been associated with beneficial metabolic profiles in humans. Serum lipids and glucose were measured in 10 week old mice after overnight fasting. Serum triglyceride, total cholesterol, and HDL were reduced significantly in *Tc1*^−/−^ mice compared to wild type controls (Fig. 3a). LDL also appeared to be lower, but not statistically significant. Fasting glucose levels were similar between *Tc1*^−/−^ and wild type mice (Fig. 3b). *Tc1*^−/−^ mice tolerated injected glucose better than wild type controls significantly. At 15 min post-injection, serum glucose rose similarly. At 30 min, however, serum glucose in *Tc1*^−/−^ mice was decreasing whereas it was still increasing in wild types. *Tc1*^−/−^ mice showed much lower serum glucose than wild types for 2 hours. No significant difference of insulin tolerance test was shown between *Tc1*^−/−^ and wild type mice (Fig. 4c).

### *Tc1* ^−/−^ ADSC proliferate actively

To investigate the mechanism of adipose hyperplasia, we isolated ADSC from inguinal sWAT from *Tc1*^−/−^ and wild type mice. Both *Tc1*^−/−^ and wild type ADSCs expressed Sca-1 and Cd44 similarly, supporting stem cell-like characters of the isolated cells (Fig. 4a). No significant Cd45 expression was present, indicating that white blood cell contamination was negligible. Upon cell cycle analysis, G2 population of *Tc1*^−/−^ ADSC was much higher than control (Fig. 4b), supporting enhanced proliferative activity of *Tc1*^−/−^ ADSC.

The proliferation of ADSC was measured using WST-1 kit. *Tc1*^−/−^ ADSC proliferated more actively compared to wild type control (Fig. 4c), which was consistent with the cell cycle analysis. The data was intriguing, because TC1 was reported to associate with active proliferation of cancer cells and its knockdown reduced the proliferative activity^21^,^27^. To investigate whether the proliferative activity was cell type-dependent, we measured the proliferation of mouse embryonal fibroblasts (MEF) similarly. In contrast to ADSC, *Tc1*^−/−^ MEF proliferated slower than wild type MEF (Fig. 4d). Together, our data supported that TC1-dependent ADSC regulation might be specific for the cell type.

### Enhanced adipogenic activity of *Tc1* ^−/−^ ADSC

The adipogenic potentials of ADSCs were investigated *in vitro*. After induction for adipogenesis*, Tc1*^−/−^ ADSCs showed robust adipose differentiation as shown by Oil Red O staining (Fig. 4e). Lipid globule containing cells were significantly increased in *Tc1*^−/−^ ADSCs than in wild type controls (Fig. 4f).

It has been shown that PPARγ and CEBPα play critical roles for adipogenesis. The expressions of PPARγ and CEBPα were not significantly different between *Tc1*^−/−^ and wild type ADSCs before adipogenic induction as measured by real-time PCR (Fig. 4g). At 24 hours post-induction, however, both PPARγ and CEBPα were robustly up-regulated in *Tc1*^−/−^ ADSCs (Fig. 4g). Our data supported that Tc1 was an upstream regulator of the major transcriptional regulators for adipogenesis. The mRNA expression of Tc1 itself did not change significantly during the induction (data not shown).

### Wisp2 and Dlk1 down-regulated in *Tc1* ^−/−^ ADSC

**Figure 5:**
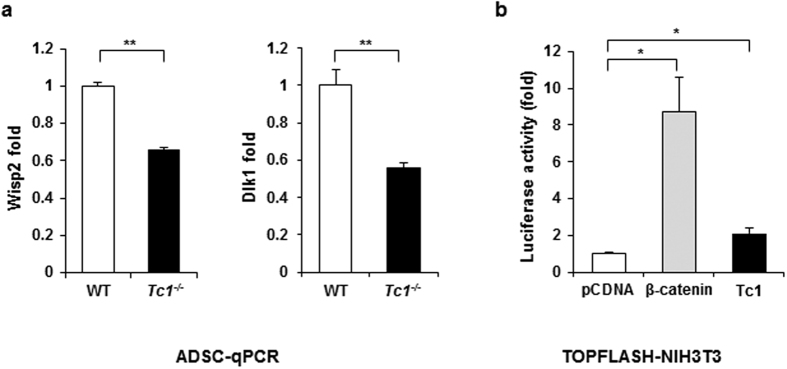
Adipogenic inhibitor gene expression and TOPFLASH analysis. (**a**) Real-time PCR for Dlk1 and Wisp2 expression in wild type and *Tc1*^−/−^ ADSCs. Data represent mean ± s.d. from 3 independent experiments. (**b**) TOPFLASH analysis of *Tc1*-transfected NIH3T3 cells as described in Materials and Methods. β-catenin and pcDNA3 empty vector transfections for positive and negative controls, respectively. Experiments are repeated in quadruplicate, and the fold changes of luciferase reporter activity are calculated after β-galactosidase normalization. **p < 0.01, ***p < 0.001.

We further investigated potential regulatory mechanisms of Tc1-dependent adipose regulation using ADSCs. Wisp2 is an adipokine secreted by adipose precursor cells to inhibit adipogenic differentiation^30^. It is one of the up-regulated genes in abdominal sWAT of humans having hypertrophic obesity^31^. Dlk1 is implicated in the restriction of adipose tissue volume by inhibiting preadipocyte cell cycle progression and proliferation^32^,^33^. Both Wisp2 and Dlk1 were significantly down-regulated in *Tc1*^−/−^ ADSCs compared to wild type controls upon real-time PCR analysis, (Fig. 5a), suggesting their roles in the hyperplastic obesity of *Tc1*^−/−^ mice at the stem cell levels.

Canonical Wnt signaling is implicated in the inhibition of adipogenesis^6^,^34^. It has been reported that TC1 enhances Wnt/β-catenin signaling in cancer cells^27^. We investigated the role of Tc1 for the signaling with TOPFLASH reporter system in NIH3T3 cell that has adipogenic stem cell-like potential. The β-catenin reporter activity was enhanced in *Tc1*-transfected cells mildly compared to vector transfected control (Fig. 5b), suggesting a potential role of canonical Wnt signaling in Tc1-dependent adipogenic regulation. The expression patterns of Wnt-downstream genes in ADSCs are inconclusive (data not shown). Wnt signaling is known to be cell context-dependent^35^.

## Discussion

Obesity-induced metabolic complications pose serious public health problems. Evidences have been accumulated to support that limited expandability of adipose tissue is directly associated with the complications whereas hyperplastic obesity has a positive protective effect. However, the regulatory mechanism for adipose tissue cellularity is poorly understood. Adequate animal models would be critical to understand the underlying biology that might provide an insight for therapeutic approaches. As far as we are aware of, *Tc1*^−/−^ mice represents the first informative animal model for spontaneous adipose tissue hyperplasia and stem cell regulation. The expansion of BAT in *Tc1*^−/−^ mice may be implicated in the enhanced metabolic regulation.

It is interesting that the adipose hyperplasia of *Tc1*^−/−^ mice is age-dependent. Young *Tc1*^−/−^ mice weigh heavier than wild type, but the weight gap decreased as they grow. sWAT adipocytes are smaller in size in both young and aged *Tc1*^−/−^ mice compared to wild type, suggesting that the adipose hyperplasia is maintained in aged animals. Our data might recapitulate the pattern of adipocyte regulation in humans. It has been reported that adipocyte number in humans augments after birth and during adolescence, but varies little during adulthood regardless of weight changes^36^. Turnover of adipocytes continues throughout life as 10% per year, but the total number of adipocytes in adulthood seems to be tightly regulated.

Our data suggest that the adipose tissue hyperplasia of *Tc1*^−/−^ mice is regulated at the stem cell level. ADSCs from *Tc1*^−/−^ mice show more proliferative activity and adipogenesis compared to wild type, supporting that Tc1 regulates adipose stem cell activity. Interestingly, the Tc1-dependent stem cell regulation appears to be cell type-dependent. In contrast to ADSCs, *Tc1*^−/−^ MEFs proliferate less actively than wild type control do. MEFs are undifferentiated mesenchymal cells that may share stem cell-like characters. In our previous study, *Tc1*^−/−^ mice showed enhanced hematopoietic activity, but no enhancement of known hematopoietic growth factors was detected in bone marrow^29^. Together with data of this study, it might be postulated that Tc1 regulates bone marrow mesenchymal stem cell, which supports not only hematopoietic activity but also diverse systemic replenishment including adipose tissue^37^. Further studies are required for systemic stem cell regulation underlying hyperplastic obesity of *Tc1*^−/−^ mice.

Tc1 regulates adipogenic downstream genes at the transcriptional level in ADSCs. PPARγ and CEBPα are up-regulated robustly in *Tc1*^−/−^ ADSCs after adipogenic induction. Negative regulators of adipogenesis such as Wisp2 and Dlk1 are down-regulated in *Tc1*^−/−^ ADSCs compared to controls, suggesting their roles in TC1-dependent adipose hyperplasia. Our data together suggest a role of Tc1 as a comprehensive upstream regulator of adipose stem cells and adipogenesis. Wnt signaling may also be involved in the regulation. *Tc1*^−/−^ mice appears to provide a unique model for adipose stem cell regulation. Underlying regulatory mechanisms for improved metabolic profile need to be investigated. Currently, we are investigating the systemic changes in *Tc1*^−/−^ mice on diet-induced obesity.

## Methods

### Mice

*Tc1*-deleted mice were described previously^29^. They had been backcrossed to C57BL/6 mice for 10 generations and kept in specific pathogen-free (SPF) animal facility at the Asan Institute for Life Sciences, Seoul, Korea. Standard chow diet was given *ad libitum* for this study. Experiments were done using *Tc1*^−/−^ of 4 to 23 weeks old male mice and age-, and sex-matched wild type controls, as indicated. All animal studies were performed with the approval by the Animal Care and Use Committee of the Asan Institute for Life Sciences following the experimental guidelines (Permit Number: 2013–12–080).

### Magnetic resonance imaging (MRI)

The MRI experiments were performed in 9.4T/160 mm Agilent MRI scanner (Agilent Technologies, Santa Clara, CA) using a millipede shape volume RF coil. *Tc1*^−/−^ and wild type control mice were anesthetized by spontaneous inhalation of 1.5–2% isoflurane using a mask, and placed in the handling system in the prone position. The parameters of T1w image were: relaxation time (TR) = 1100 ms, kzero = 1, segments/echo train length (ETL) = 48/4, effective TE = 9.78 ms, 4 averages, matrix = 192 × 192, the field of view (FOV) = 25 × 35 mm, slice thickness = 2.0 mm, and total scan time = 4 min 24 sec, respectively. During MR scanning, the external triggering was used to eliminate the respiratory motion artifacts. For MRI fat volumetry, Image J software (National Institutes of Health, MD, http://rsb.info.nih.gov/ij/) was used. The volume of subcutaneous white adipose tissue (sWAT) and visceral white adipose tissue (vWAT) were calculated in mm^3^ by sum of the regions multiplied by slice thickness, respectively.

### Adipose tissue analysis

sWAT, vWAT, and BAT of male *Tc1*^−/−^ mice and age- and sex-matched wild type were dissected separately (6 week old, n = 6 each group; 10 week old, n = 8 each group; 23 week old, n = 5 each group, respectively). The percentage per whole body weight was calculated for every adipose tissue samples and lean body mass. For histological analysis, tissue slices were fixed in buffered formalin and paraffin-embedded. Histological sections of 4 μm thickness were cut and stained for hematoxylin and eosin. Images were acquired using an Olympus BX53 microscope connected to an Olympus DP73 digital camera. Histological images were analyzed to calculate the average size of adipocytes using ImageJ Software. For the analysis, 10 random images were captured for every sample.

### Serum glucose and lipid profile

*Tc1*^−/−^ and wild type mice were fasted overnight for 15 h. Under deep anesthesia, blood samples were taken from the inferior vena cava, and serum samples were collected by centrifuging at 3000 rpm. for 15 min. Glucose, triglyceride, total cholesterol, low-density lipoprotein (LDL), and high-density lipoprotein (HDL) were analyzed by the Green Cross Laboratories (Kyoungi-Do, Korea).

### Glucose and insulin tolerance test

For glucose tolerance test, 10 week old *Tc1*^−/−^ and control mice were fasted overnight (n = 6 each). They were injected with 2% glucose solution, 2 g/kg body weight, intraperitoneally. Blood samples were taken from mouse tail veins at 15, 30, 45, 60, 90 and 120 min after the injection, and blood glucose was measured using ACCU-CHEK^®^ (Roche Diagnostics, Seoul, Korea). For insulin tolerance test, 10 week old *Tc1*^−/−^ and control mice (n = 4) were fasted for 5 hours. Recombinant insulin (Humulin, Eli Lily), 1unit/kg, was injected intraperitoneally. Blood glucose was measured similarly.

### ADSC isolation and proliferation assasy

Inguinal sWAT was dissected from *Tc1*^−/−^ and wild type mice, minced and digested using 0.5% collagenase (Sigma Aldrich, St. Louis, MO) at 37 °C for 1 h. Cell suspensions were filtered through a cell strainer with 100 μm nylon mesh (BD Biosciences, San Jose, CA), washed twice with phosphate-buffered saline (PBS) by centrifugation for 3 min at 1,500 rpm at 4 °C. Cell pellets were suspended in red blood cell lysis buffer (Sigma Aldrich) and incubated for 5 min. Cells were washed and centrifuged twice in PBS, and incubated in Dulbecco’s modified Eagle’s medium (DMEM, Gibco, Carlsbad, CA) with 10% fetal bovine serum (FBS, Gibco), and 1% penicillin/streptomycin (Life Technologies, Carlsbad, CA) at 37 °C in 5% CO_2_. The media were replaced after 24 h, and every 3 days thereafter. Cultures were incubated for 4~7 days until 70~80% confluence was attained. ADSCs were passaged and replated at initial concentration of 5 × 10^5^ cells per 100 mm dish. All experiments performed using passage 2 ADSCs. Cell proliferations were analyzed using WST-1 proliferation assay kit according to the manufacturer’s instruction (Roche, Mannheim, Germany). In each experiment, 4 × 10^3^ cells/well were plated in 96-well plates, and the proliferation was measured in quadruplicate at 0, 24, 48, 72, and 96 h.

### Mouse embryonal fibroblast (MEF) culture

MEFs were isolated from 13.5 day old embryos obtained from *Tc1*^−/−^ and wild type mice, respectively. After removal of head and internal organs, embryos were dissociated and then trypsinized to produce single-cell suspensions. MEFs were expanded in modified Eagle’s medium (MEM, Gibco), and frozen in aliquots. The proliferation was measured using WST-1 proliferation assay as described.

### *In vitro* adipogenesis of ADSCs

ADSCs were plated in 6-well cell culture plates, 7 × 10^4^ cells per well. After 48 h at 70~80% confluence, the medium was changed to adipogenic induction medium (DMEM, 10% FBS, 1 μM dexamethasone, 5 μg/ml insulin, 500 nM IBMX, 50 μM indomethacin, and 1% penicillin/streptomycin). The medium was changed every 3 day thereafter for 11 days. For Oil Red O staining, cells were washed with PBS, fixed with buffered 4% formaldehyde for 20 min, rinsed with distilled water, and stained with Oil Red O solution for 10 min. Oil Red O solution was prepared before staining by diluting the stock solution (Sigma-Aldrich) in distilled water (3:2 v/v). Positive cells in 20 grids of 1 mm^2^ size were counted per well using an inverted microscope by two observers independently.

### Flow cytometry

Single-cell suspension was prepared in ice-cold PBS containing 5% FBS, and 10^6^ cells were analyzed with 1 μg antibody using FACSCantoII (BD Biosciences) flow cytometer. FITC-conjugated Sca-1 (BD Biosciences, Franklin Lakes, NJ), PE-conjugated Cd44 (BD Biosciences), and APC/Cy7-conjugated Cd45 (Bioregend, San Diego, CA), were used as indicated. FACSDiva software (BD Biosciences) and FlowJo (Tree Star) were used for data acquisition and analysis. For the cell cycle analysis, 10^6^ cells were fixed in 1 ml PBS with 50% ethanol on ice for 1 h. Cells were resuspended in 0.25 ml of PBS with 50 μg RNase (DNase free) and incubated at 37 °C for 1 h. After incubation with 10 μg/ml propidium iodide on ice in dark, cell cycle analysis was performed using a single-parameter histogram with linear x-axis to represent DNA content.

### Quantitative RT-PCR

Total RNA was extracted using Trizol reagent (Invitrogen, Carlsbad, CA), and cDNA was synthesized using Premium reverse transcriptase (Thermo Scientific, Marietta, OH). qPCR was performed using a continuous fluorescence detecting thermal cycler ABI PRISM^®^ 7000 Sequence Detection System (ABI, Foster city, CA), and a SYBR^®^ Green real-time PCR master mix (Toyobo, Osaka, Japan). Measurements were done in triplicate using β-actin as endogenous control. PCR primers were as follow: Tc1 5′-ACCAGCATGTCCTCGTCTCT-3′ and 5′-ATGTTGCCCACAGCTTTCTT-3′; Pparγ 5′-CGAGAAGGAGAAGCTGTTGG-3′ and 5′-GAAACTGGCACCCTTGAAAA-3′; Cebpα 5′-TTACAACAGGCCAGGTTT-3′ and 5′-CTCTGGGATGGATCGATT-3′; Wisp2 5′-CTGGTTTGTCAGCCTGGG-3′ and 5′-CATCACAGCGGCACAAAAC-3′; Dlk2 5′-TCAATGGAGTCTGCAAGGAA-3′ and 5′-ATTCGTACTGGCCTTTCTCC-3′; and β-actin 5′-GATCATTGCTCCTCCTGAGC-3 and 5′-ACATCTGCTGGAAGGTGGAC-3′.

### TOPFLASH analysis

NIH3T3 cells were transiently transfected in 6 well plates using Lipofectamine3000 (Invitrogen). 8X TOPFLASH reporter (0.5 μg), β-galactosidase (0.5 μg), Flag-β-catenin (2 μg), HA-Tc1 (2 μg), and/or empty vector pcDNA3 (2 μg) were applied. Expression vectors were described previously^27^. After 36 hours, the luciferase activity was measured in cell lysates. Experiments were done in quadruplicate, and the fold changes were calculated after normalization on β-galactosidase activity for transfection efficiency.

### Statistical Methods

All measurements are presented as the mean ± s.d. Statistical analyses were performed by two-tailed *t* -tests or ANOVA tests using SPSS version 20 (SPSS Inc., Chicago, IL).

## Additional Information

**How to cite this article**: Jang, H. *et al*. Adipose tissue hyperplasia with enhanced adipocyte-derived stem cell activity in *Tc1*(*C8orf4*)-deleted mice. *Sci. Rep.* **6**, 35884; doi: 10.1038/srep35884 (2016).

## Supplementary Material

Supplementary Information
