# Supplementary material for: Adipose tissue hyperplasia with enhanced adipocyte-derived stem cell activity in Tc1(C8orf4)-deleted mice
Source: Sci Rep. 2016 Oct 24;6:35884. doi: 10.1038/srep35884 (PMC5075883; doi:10.1038/srep35884)
Supplement: Supplementary Information [file srep35884-s1.doc]

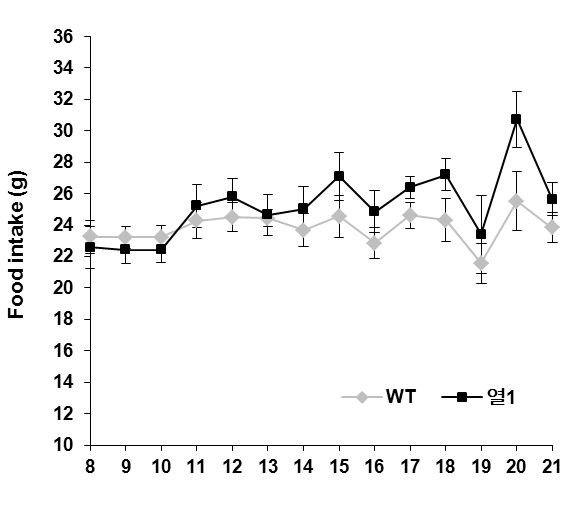


**wk**

***Tc1*^-/-^**

**WT**

**wk**

**Food intake /mouse /week**

**(mean ± s.d. of 9 male *Tc1*^-/-^ and wild type mice )**

**Supplemental Figure S1**

Adipose tissue hyperplasia with enhanced adipocyte-derived stem cell activity in *Tc1*(*C8orf4*)-deleted mice

Jang H, Kim M, Lee S, Kim J, Woo D, Kim K, Song K, Lee I

**Whole body Lean mass**

**
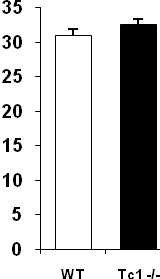
** **
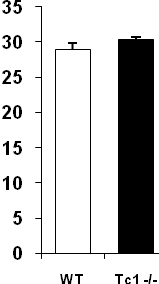
**

**(g)**

**WT *Tc1^-/-^***

**WT *Tc1^-/-^***

**WT *Tc1^-/-^***

**WT *Tc1^-/-^***

**BAT sWAT vWAT**

**
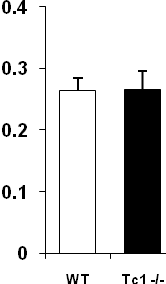
** **
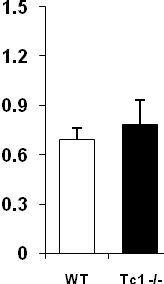
** **
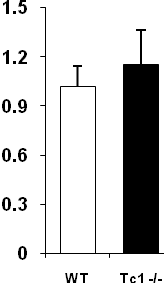
**

**(g)**

**WT *Tc1^-/-^***

**Whole body, lean body mass, BAT, sWAT, and vWAT weights**

**23 week old, male *Tc1^-/-^* and wild type mice.**

**(mean ± s.d. of 8 animals each group)**

**Supplemental Figure S2**

Adipose tissue hyperplasia with enhanced adipocyte-derived stem cell activity in *Tc1*(*C8orf4*)-deleted mice.

Jang H, Kim M, Lee S, Kim J, Woo D, Kim K, Song K, Lee I
